# Mapping Mutations and Signaling Network Interactions to Guide Precision Therapy in Gallbladder Cancer

**DOI:** 10.1002/cnr2.70699

**Published:** 2026-09-30

**Authors:** Nisha Manav, Akanksha Kashyap, Lakshay Malhotra, Chethan Rajegowda, Chandra Prakash Prasad

**Affiliations:** ^1^ DR. B.R.A. IRCH AIIMS New Delhi India; ^2^ Department of Biochemistry Sri Venkateswara College, University of Delhi New Delhi India

**Keywords:** gallbladder cancer, mutations, oncogene, oncogenic signalling, therapeutics, tumor suppressor proteins, tumorigenesis

## Abstract

**Background:**

Gallbladder cancer (GBC) is a biologically complex malignancy arising from the epithelial lining of the gallbladder, with adenocarcinoma constituting the major histological subtype. Early detection is challenging because of vague clinical manifestations and the organ's deep‐seated anatomical location, resulting in diagnosis at advanced stages and limited treatment options. Consequently, patients often experience poor prognosis with low 5‐year survival rates. Clinical and translational studies have demonstrated marked biological heterogeneity in GBC, primarily driven by diverse molecular alterations that fuel tumor initiation and progression.

**Methods:**

In the present study, we investigated recurrently mutated genes in GBC using The Cancer Genome Atlas (TCGA) and Catalogue of Somatic Mutations in Cancer (COSMIC) gallbladder cohorts, focusing on genes with a mutation frequency above a pre‐specified threshold (≥ 5% in at least one dataset). We then cross‐referenced these genes with published literature till date in GBC and other solid tumors where the same variants have been functionally characterized as loss‐of‐function or gain‐of‐function. “Likely loss of function” was assigned only when mutations were annotated in OncoKB/COSMIC as truncating/nonsense or as missense variants with experimental or strong computational evidence of functional impairment.

**Results:**

Evaluation and the functional impact of prominent mutations viz. TP53, SMAD4, PIK3CA, CDKN2A, ARID1A, ARID2, KRAS, ELF3, ERBB3, ERBB2, STK11, and CTNNB1 on key cellular signaling pathways that regulate GBC initiation and progression. These alterations influence various oncogenic mechanisms, including cell proliferation, survival, apoptosis evasion, and metastatic potential, by disrupting pathways. By comparing the mutational patterns observed in GBC with those reported in other malignancies sharing analogous molecular signatures, we delineate potential therapeutic vulnerabilities.

**Conclusions:**

Mutation‐guided therapeutic approaches hold great promise for advancing precision medicine in GBC. By tailoring treatments to the specific molecular alterations driving tumor development, this could pave the way for more effective interventions and improved patient outcomes in a disease long constrained by limited therapeutic options.

## 1 Introduction

Gall bladder cancer (GBC) is the most prevalent malignancy of the biliary tract, originating from the epithelial lining of the gall bladder, affecting a considerable portion of the population every year [1]. Among all its histological subtypes, adenocarcinoma accounts for nearly 90% of GBC cases, while the less frequently reported subtypes include undifferentiated, squamous cell, mucinous, and neuroendocrine carcinomas, each associated with distinct morphological and clinical characteristics [2]. GBC often remains clinically silent during its early course due to its deep anatomical location and nonspecific manifestations. This insidious progression, combined with its highly aggressive metastatic behavior, frequently leads to diagnosis at advanced stages, where therapeutic interventions are limited and patient outcomes are poor, resulting in reduced survival rates [3]. Consequently, survival outcomes remain dismal: while early‐stage GBC patients may achieve a 5‐year survival rate of 30%–40%, those diagnosed with locally advanced lesions face a markedly reduced 1‐year survival rate of approximately 10% [4, 5]. Even after surgical resection, recurrence rates remain high, with 60%–70% of patients experiencing disease relapse [6, 7]. Epidemiologically, the incidence of GBC increases with age. Women are 2–6 times more susceptible than men, and this disease is more prevalent among individuals of caucasian descent compared to those of African ancestry [4].

In this review, we identified the most frequently mutated proteins in GBC patient dataset using The Cancer Genome Atlas (TCGA) and COSMIC database and the possible outcome of their mutation on the signaling crosstalk that drives tumorigenesis and progression of GBC. By integrating insights from other malignancies with similar molecular aberrations, we outline a potential path forward for therapeutic strategies aimed at targeting these mutant proteins. This approach may offer promising avenues for precision oncology in GBC, addressing the urgent need for effective targeted therapies in cancer types where conventional treatment options remain limited and clinical outcomes are poor.

### 1.1 Epidemiological Trends and Demographic Vulnerabilities in GBC

According to GLOBOCAN 2022, GBC accounted for a total of 122 491 new cases globally, with a notable gender disparity: 43536 cases in males and 78 933 cases in females [8]. This malignancy also led to 89 055 fatalities, comprising 31 402 males and 57 643 females. Despite being relatively rare in global cancer statistics, accounting for 1.3% of all the global cancer incidence and 1.7% of the cancer‐related mortalities, it poses a significant health burden due to its aggressive and poor prognosis [8].

Epidemiological studies of GBC reveal a pattern of geographic variability of its occurrence, with Asia being the hotspot, accounting for approximately 71% and 75% of the incidence and mortality cases, respectively (Figure 1a). Within Asia, countries like China and India are the epicentre of GBC cancer, responsible for 25.4% and 17.8% incidence cases and 27.6% and 18.4% of GBC‐related mortality cases, respectively. However, the Age‐standardized rate of incidence per 100 000 humans (ASR) of GBC reveals that countries like Chile, Bolivia, Peru, and Argentina in South America; Algeria and Libya in North Africa; Poland and Czech in Europe; Pakistan, India, China, Nepal, Bangladesh, and Myanmar in South East Asia have very high frequencies of GBC incidence as compared to other global areas (Figure 1b) [9].

**FIGURE 1:**
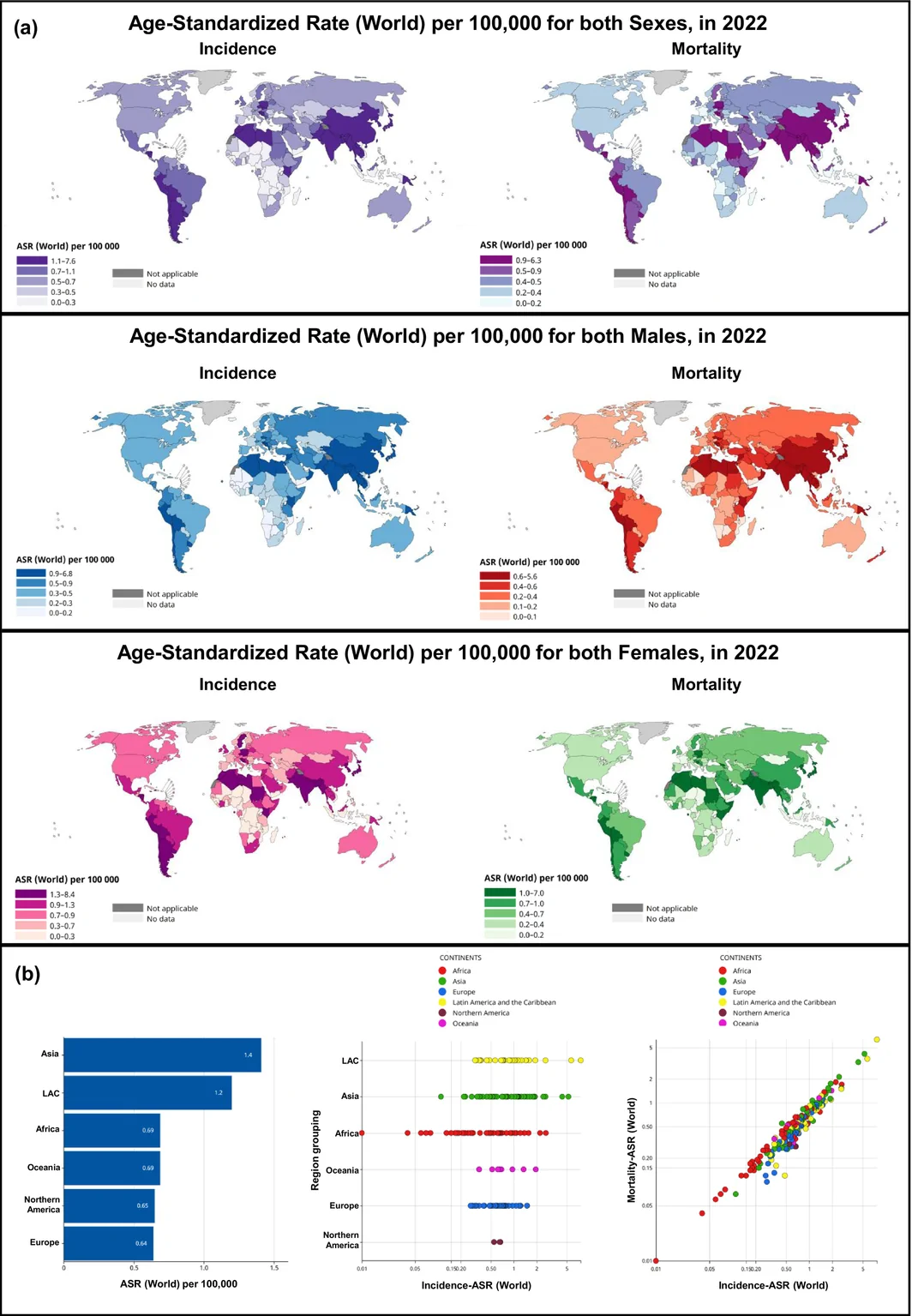
Global burden of gallbladder cancer based on GLOBOCAN 2022 data: (a) Age‐standardized incidence and mortality rates (ASR per 100 000) for gallbladder cancer, shown globally for both sexes combined, males alone, and females alone. Incidence rates are displayed on the left, and mortality rates on the right. (b) Global (continental) distribution of ASR per 100 000 (left); regional grouping of incidence ASR (middle); and scatter plot comparing mortality ASR versus incidence ASR across world regions (right). Data adapted from GLOBOCAN 2022.

Population‐based studies in India have revealed a distinct geographic clustering of GBC cases with markedly higher incidence rates in some specific areas [5]. It was observed that the frequency of GBC incidence risk in North and North‐eastern India is nearly seven times greater than in Southern India, positioning states like Bihar, Uttar Pradesh, Assam, Orissa, and West Bengal as the primary hotspots. The ASR reported from these states is tantamount to the high‐prevalence regions of the world [10, 11]. This regional disparity in GBC incidence across ethnicities, cultures, and geographies is likely driven by a multifactorial interplay of physiological, genetic, and environmental risk factors contributing to GBC pathogenesis. Gender‐based analysis of GBC epidemiological analysis further underscores a significant susceptibility among women, who are nearly twice as susceptible as men to develop GBC and succumb to GBC [8, 12, 13]. Among female patients, high parity and early age at first pregnancy have been identified as notable risk enhancers for GBC [14].

Etiological factors such as advanced age (median 50–55 years), a positive family history of GBC, cholelithiasis, gall bladder polyps, and chronic infections with *H. pylori* and *S. typhi* have been consistently associated with GBC onset [15]. In parallel, lifestyle‐related risks such as tobacco and alcohol consumption, obesity, and prolonged exposure to harmful chemicals and pollutants like aflatoxins and arsenic further exacerbate GBC susceptibility. These factors often act synergistically with genetic predisposition, particularly somatic mutations in key oncogenic and tumor suppressor genes, contributing to the multistep carcinogenesis of GBC [15].

## 2 Mutational Landscape of GBC

While a multitude of risk factors, including environmental exposures and lifestyle determinants, contribute to the likelihood of developing GBC, it is the molecular alterations in oncogenes and tumor suppressor genes that serve as pivotal drivers of its pathogenesis. Additionally, the microbial dysbiosis also creates a carcinogenic environment that accelerates mutation accumulation. These genetic aberrations, ranging from point mutations and copy number variations to epigenetic modifications, collectively shape the mutational landscape of GBC and critically influence its clinical behavior and trajectory.

Before focussing into the mutational landscape of gallbladder cancer (GBC), it is essential to consider a central driver of its pathogenesis that is, microbial dysbiosis within the biliary and gastrointestinal ecosystems. Accumulating evidence positions dysbiosis as a pivotal force in GBC initiation and progression, fostering a pro‐tumorigenic milieu through chronic inflammation, reprogramming of bile acid (BA) metabolism, and heightened genotoxic stress conditions that collectively facilitate the stepwise accrual of oncogenic mutations and the transition from chronic cholecystitis to dysplasia and invasive carcinoma [16]. Dysbiosis promotes carcinogenesis via multiple, intersecting mechanisms that elevate mutational burden and enable clonal expansion. A hallmark feature is the enrichment of taxa such as Enterobacteriaceae, Streptococcus, and Helicobacter species, which drive a metabolic shift characterized by enhanced microbial 7*α*‐dehydroxylation of primary BAs, cholic acid (CA) and chenodeoxycholic acid (CDCA) into secondary BAs, including lithocholic acid (LCA) and notably deoxycholic acid (DCA) [16]. DCA and related metabolites exert detergent‐like, genotoxic effects: they inflict DNA damage, activate pro‐survival and proliferative signaling cascades (e.g., EGFR, Wnt/*β*‐catenin), and perpetuate cycles of epithelial injury and regeneration, each cycle increasing the probability of mutation fixation. Concurrently, chronic inflammation and reactive oxygen species (ROS) production, often amplified by specific bacterial pathogens (e.g., Helicobacter spp.), directly contribute to TP53 mutations, the most frequent genomic alteration in GBC. Pathogens including *Helicobacter hepaticus* , *Salmonella Typhi* , *S. paratyphi* A, *Klebsiella pneumoniae* , other Enterobacteriaceae, and *Helicobacter pylori* secrete virulence factors and genotoxins such as cytolethal distending toxin (CDT), colibactin, and DNA‐binding proteins that trigger DNA damage responses, induce genome instability, and ultimately promote malignant transformation. Genomic profiling of GBC has identified recurrent mutations in TP53, SMAD4, NOTCH1, and ERBB2, underscoring the convergence of microbial‐driven genotoxicity and canonical oncogenic pathways [17]. Thus, microbial dysbiosis in GBC is not merely a bystander phenomenon; it actively sculpts a microenvironment that accelerates both the generation and selection of oncogenic mutations, thereby influencing tumor evolution and therapeutic vulnerability.

Given the central role of these molecular events, a comprehensive understanding of the genetic architecture of GBC is imperative. Such insights not only illuminate the underlying mechanism of tumorigenesis and progression but also facilitate the discovery of novel early diagnostic biomarkers and actionable therapeutic targets [18, 19]. A more nuanced elucidation of GBC's mutational architecture and dynamics will be instrumental in advancing precision oncology approaches and refining prognostic satisfaction for improved patient outcomes.

By leveraging comprehensive genomic profiling using the Catalogue of Somatic Mutations in Cancer (COSMIC), correlated with TCGA databases, several genes have been identified as most frequently mutated and functionally significant in GBC (Figure 2a). These recurring mutations not only underscore the molecular heterogeneity of the disease but also pinpoint critical nodes of dysregulation within tumor suppressor networks and oncogenic signalling cascades. Globally, the most prominent contributors to GBC pathobiology are TP53, SMAD4, PIK3CA, CDKN2A, ARID1A, ARID2, KRAS, ELF3, ERBB3, ERBB2, STK11 and CTNNB1, each offering distinct, valuable insights into the mechanisms of malignant transformation and potential avenues for targeted intervention. In Indian cohorts, TP53 emerged as the predominant driver mutation, while ERBB2 was identified as a clinically actionable target. Conversely, ARID1A, SMAD4, and CDKN2A mutations were detected at lower frequencies relative to global reports [20] (Figure 2b). These proteins contribute to GBC pathogenesis through regulation of critical cellular signaling pathways, including PI3K/AKT and the cell cycle machinery (Figure 2c). Mutations affecting these proteins disrupt their normal regulatory roles, leading to aberrant activation or suppression of downstream signaling cascades. This dysregulation results in uncontrolled proliferation, impaired apoptosis, and additional oncogenic traits, ultimately driving the malignant phenotype observed in GBC. A lollipop plot illustrating the most frequent mutations identified in each of the major proteins discussed, as observed in GBC, is presented in Figure 2d.

**FIGURE 2:**
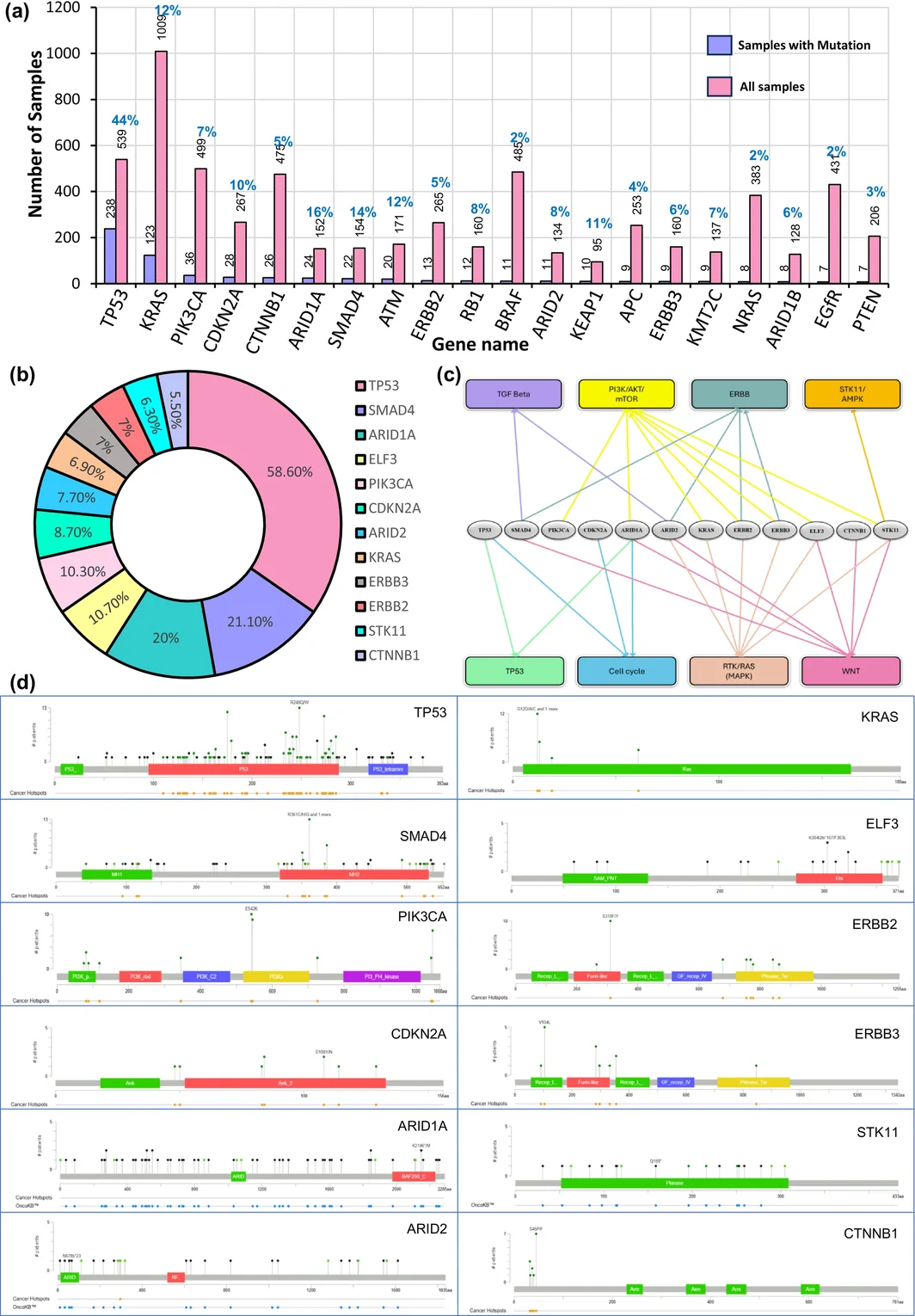
Distribution of the most frequently mutated genes in gallbladder cancer (GBC): (a) Bar graph depicting the top 20 mutated genes reported in the COSMIC database. Pink bars represent the total number of tumor samples analyzed, while purple bars indicate the number of samples harboring mutations in the respective gene. The percentage values above each bar denote the mutation frequency for each gene, calculated as the proportion of mutated samples relative to the total. (b) Doughnut chart depicting the mutation frequency (%) of genes identified in TCGA datasets that also overlap with those reported in the COSMIC database. (c) Schematic representation of signaling crosstalk involving 12 key genes frequently mutated in GBC, mapped onto major cellular pathways in a normal cell. The diagram highlights their putative roles in maintaining cellular homeostasis and regulatory functions under physiological conditions. (d) Lollipop diagram illustrating the positional distribution of the most frequently occurring mutations within the protein‐coding regions of 12 highly mutated genes in GBC, based on TCGA dataset analysis. Each lollipop represents a mutation event mapped to its corresponding amino acid position, highlighting mutation hotspots and potential functional domains.

### 2.1 TP53: The Central Tumor Suppressor Turned Oncogene in GBC

TP53, widely recognized as ‘Guardian of the Genome’, is a vital tumor suppressor protein that safeguards cellular integrity in response to a wide spectrum of physiological, chemical, and genotoxic stresses. In its wild‐type form, p53 orchestrates a multifaceted defense against internal and external stresses, such as oncogenic activation, DNA damage, hypoxia, and nutrient deprivation by regulating cell cycle arrest, DNA repair, senescence, and programmed cell death (i.e., apoptosis). These coordinated responses collectively preserve genomic stability and normal cellular homeostasis, thereby preventing malignant transformation [14, 21].

Loss of TP53 activity results in the survival and growth of genetically mutated cells, which becomes a significant event in GBC tumorigenesis. The most common mechanism involves missense mutations within the DNA‐binding domain (DBD), particularly spanning from exon 5 to 8, which impair the transcriptional activity of p53 [22, 23]. Besides the loss‐of‐function phenotype, these mutations also confer gain‐of‐function properties, resulting in the accumulation of stable, transcriptionally inactive p53 proteins. These mutated p53 proteins are markedly overexpressed in invasive GBC tissues, being virtually absent in normal Gallbladder epithelium. Hence, enhanced expression of mutated p53 serves as an early event in the neoplastic conversion of precancerous lesions [24].

Multiple studies have reported that TP53 mutations occur in up to 70% of GBC cases [14]. TCGA and the COSMIC further corroborate this, revealing mutation frequencies of 58.6% and 44%, respectively. These findings firmly establish TP53 as the most frequently mutated gene in GBC, underscoring its central role in the molecular pathogenesis of the disease and its potential as a diagnostic and therapeutic target.

### 2.2 SMAD4: A Key Mediator of TGF‐*β* Signalling in GBC

SMAD4 is a transcription factor that plays a critical role in the TGF‐*β* signaling pathway, which governs essential cellular processes such as cell proliferation, migration, and differentiation, and apoptosis [25]. Functioning as a tumor suppressor gene, SMAD4 has been implicated in the pathogenesis of various cancers, including GBC [26].

TCGA genomic cohort analysis revealed SMAD4 mutations in approximately 21% of the GBC samples, a bit higher from the datasets of the COSMIC database that report a mutation frequency of around 12%. The majority of the missense mutations are localized to the MH2 domain of the SMAD4 protein, with a smaller subset affecting the MH1 domain (as depicted in the figure). The MH1 domain mediates DNA binding and transcriptional activity, while the MH2 domain facilitates complex formation with Receptor‐regulated SMADs (R‐SMADs), essential for downstream signalling [26]. Disruption of SMAD4 impairs canonical TGF‐*β* signalling, thereby promoting tumor progression and enhancing metastatic potential in GBC [27, 28]. Because of its mutation profile and functional relevance, SMAD4 acts as a significant molecular determinant in GBC pathobiology and a candidate for therapeutic intervention.

### 2.3 PIK3CA: A Driver of Oncogenic PI3K/AKT Signaling in GBC

PIK3CA encodes the catalytic subunit p110*α* of Phosphatidylinositol 3‐kinase (PI3Ks), a family of lipid kinases that play a central role in regulating various cellular phenotypes such as cell survival, proliferation, and other metastatic properties of cells [29]. As a well‐established oncogene, PIK3CA is frequently found hyperactivated across various cancers, including GBC, where its aberrant signalling promotes tumor progression and resistance to apoptosis [30].

Comprehensive genomic profiling from the TCGA GBC cohort revealed PIK3CA mutations in approximately 10% of GBC cases, closely aligning with the data from the COSMIC database, which reports a mutation frequency of ~7% in GBC. The most recurrent and functionally significant mutations are localized to exon 9 (E542K and E545K) and exon 20 (H1047R), which are known to enhance kinase activity and constitutively activate the PI3K/AKT signaling axis [31, 32]. This persistent activation drives oncogenic signaling cascades that support cellular transformation, survival under stress, and therapeutic resistance, highlighting PIK3CA's role as a critical molecular target in GBC pathobiology.

### 2.4 CDKN2A: The Sentinel of the Cell Cycle Regulation

CDKN2A is a tumor suppressor gene and is often regarded as the sentinel of cell cycle regulation. It encodes two distinct proteins, p14 (ARF) and p16 (INK4a), both of which play key roles in maintaining cell cycle checkpoints and genomic integrity [33]. p16 protein prevents interaction between cyclin D and CDK4, thereby preventing phosphorylation of retinoblastoma (Rb) protein at residues S807 and S811. This inhibition effectively halts the G1‐to‐S phase transition, enforcing cell cycle arrest. Alongside, the p14 protein is involved in stabilization of tumor suppressor p53, through inhibition of its negative regulator MDM2 [34], thereby enhancing p53‐mediated responses such as cell cycle arrest and apoptosis. In GBC, CDKN2A mutations disrupt these regulatory mechanisms, contributing to uncontrolled cell proliferation. The TCGA GBC cohort analysis revealed CDKN2A mutations in approximately 9% of cases, which closely aligned with COSMIC data, indicating a mutation frequency of 10%. According to OncokB annotations, these CDKN2A mutations are classified as either likely oncogenic or oncogenic, primarily due to loss‐of‐function alterations that compromise cell cycle control [35], thus leading to uncontrolled tumor growth, thereby making it another promising therapeutic target.

### 2.5 ARID1A: A Chromatin Remodeler Turned Oncogene

AT‐Rich Interaction Domain 1A (ARID1A) is a key subunit of the SWItch/Sucrose Non‐Fermentation (SWI/SNF) remodeling complex, involved in transcription regulation, DNA damage response, and signal transduction [36]. It functions as a tumor suppressor and is frequently mutated in GBC.

TCGA cohort analysis reveals ARID1A mutations in ~20% of tumour samples, whereas the Cosmic database highlights a 16% mutation frequency in GBC patients. The majority of these mutations were found to be nonsense or truncating mutations, resulting in the formation of truncated proteins. Oncokb database annotates all ARID1A substitutions mutations as likely oncogenic with likely loss of function. Mutated ARID1A enhances proliferative, migratory, and invasive phenotypes [37]. A study by Nan et al. demonstrated that ARID1A deficiency leads to the overexpression of PD‐L1 and impaired function of tumour‐infiltrating lymphocytes (TILs), facilitating immune evasion and a poor prognosis [38]. These alterations correlate with reduced overall survival, underscoring the clinical significance of ARID1A.

### 2.6 ARID2: A PBAF Complex Subunit Driving Chromatin Dysregulation in GBC

AT‐Rich Interaction Domain 2 (ARID2) is another important subunit of the SWItch/Sucrose Non‐Fermentation (SWI/SNF) remodelling machinery, specifically within the polybromo‐associated BAF (PBAF) subclass [39]. It plays a pivotal role in nuclear receptor‐mediated, ligand‐dependent transcriptional activation by modulating chromatin accessibility, thereby facilitating gene expression in response to cellular signals [40].

Like ARID1A, ARID2 is a tumor suppressor gene which is recurrently mutated in GBC, with the mutation frequencies reported at approximately 8% in both TCGA and COSMIC cohorts. These mutations, primarily loss‐of‐function variants, are annotated by Oncokb database as likely oncogenic due to their disruptive impact on chromatin remodeling and transcriptional regulation. Loss of ARID2 function compromises the structural integrity of the PBAF complex, impairing its ability to regulate gene expression and maintain genomic stability. This dysregulation contributes to tumorigenesis by promoting aberrant transcriptional programs, cellular proliferation, and potentially immune evasion [39]. Like ARID1A, ARID2 mutations emphasize the significance of chromatin remodelling defects in GBC pathobiology and represent a promising avenue for therapeutic exploration.

### 2.7 KRAS: A Proto‐Oncogene Driving Aberrant RAS Signalling in GBC

Kirsten rat sarcoma (KRAS) is a key proto‐oncogene that encodes a small GTPase functioning as a molecular switch involved in the RAS–RAF–MEK–ERK signalling cascade. This pathway governs essential cellular events such as cell proliferation, differentiation, and survival [41]. KRAS cycles between an active GTP‐bound state and an inactive GDP‐bound state, tightly regulating downstream signalling events. In GBC, mutations in KRAS lead to constitutive activation of the protein, resulting in persistent proliferative signalling and abnormal tumorigenesis. In both TCGA and COSMIC databases, approximately 7%–12% of the GBC cases have been reported to have KRAS mutations. The most prevalent mutations in KRAS are observed at codon 12, with base substitutions such as G15C, G12D, and G12V [42]. These mutations impair GTP hydrolysis, locking KRAS in its active state and driving oncogenic signalling.

Given its role in promoting unchecked cellular proliferation and its relatively high mutation frequency, KRAS represents a critical molecular target in GBC. Emerging therapeutic strategies targeting KRAS G12 mutation variants may offer promising avenues for precision oncology in this subset of patients.

### 2.8 ELF3: A Context‐Dependent Regulator With Tumor Suppressive Role in GBC

E74‐like factor 3 (ELF3) is a member of the E‐twenty‐six (ETS) transcription factor family, known for regulating gene expression linked to cellular proliferation, migration, and invasion [43]. ELF3 exhibits context‐dependent roles across different cancers, functioning either as a tumor suppressor or oncogene depending on the tissue type and microenvironment [44].

In non‐small cell lung cancer, ELF3 has been characterized as an oncogene, promoting cellular growth and metastasis via modulation of PI3K/Akt and ERK pathways [45]. Conversely, in GBC, ELF3 functions as a tumor suppressor. A study by Nakamura et al. demonstrated that ELF3 deletion enhances epithelial‐to‐mesenchymal transition (EMT) and tumor progression through upregulation of epiregulin (Ereg), leading to activation of EGFR/mTORC1 signaling in GBCs [46]. TCGA genome analysis reveals ELF3 mutations in approximately 10% of GBC cases. Moreover, reduced ELF3 expression correlated with advanced tumor stage, increased invasiveness, and poorer overall survival, highlighting its prognostic significance. Altogether, this dual nature of ELF3 emphasizes the importance of tumor‐specific context in interpreting transcriptional regulators and opens avenues for targeted modulation in GBC therapeutics.

### 2.9 ERBB2 and ERBB3: Oncogenic Receptor Tyrosine Kinases Driving PI3K/AKT Activation in GBC

ERBB2 (Her2/neu) and ERBB3 (Her3) belong to the ERBB tyrosine kinase receptor family, a crucial receptor family in driving the process of tumorigenesis through various cellular processes such as proliferation, survival, growth and apoptosis, etc. Full activation of ERBB3 requires its phosphorylation by ERBB2. The heterodimer complex of ERBB2 and ERBB3 primarily activates the PI3K/AKT oncogenic pathway to promote tumorigenesis. Apart from being overexpressed in various cancers including GBC, these are also reported to be mutated at very significant frequencies [47]. TCGA and COSMIC data analysis reveal 5%–8% and 6% mutation frequency of ERBB2 and ERBB3 in GBC samples, respectively. Moreover, as per the oncokb database, these mutations are implicated to be either likely oncogenic or oncogenic due to likely gain of function or gain of function. A study by Li et al. revealed that ERBB2/ERBB3 mutants robustly activate the PI3K/AKT pathway, driving GBC progression in vivo [48]. Furthermore, these mutations are associated with poor clinical outcomes, underscoring their prognostic significance [49].

### 2.10 STK11: Tumor Suppressor Kinase Disrupted in GBC

Serine Threonine Kinase 11 (STK11), a key tumor suppressor, is a kinase involved in the AMPK signaling pathway. It exerts pleiotropic roles in cancer pathogenesis by regulating cellular proliferation, growth, metabolism, and metastasis through transcription as well as translation control of a plethora of downstream targets [50].

STK11 is recurrently found mutated in various cancers, including GBC. TCGA Genome analysis revealed a mutation frequency of approximately 9% in the STK11 protein. These mutations predominantly affect the kinase domain, impairing its noble functional activity and downstream signalling. Functionally, STK11 mutations are characterized to be oncogenic due to the loss‐of‐function effects and are correlated with poor patient prognosis.

### 2.11 CTNNB1: *β*‐Catenin Mutations Driving Aberrant Wnt Signaling in GBC

CTNNB1 encodes *β*‐catenin, a key cytoplasmic protein involved in the canonical Wnt signaling pathway. Under basal conditions, *β*‐catenin is tightly regulated through phosphorylation at N‐terminal serine/threonine residues within exon 3 region by upstream kinases such as GSK3*β* and casein kinase‐1 (CK‐1), marking it for proteasomal degradation. However, Wnt pathway activation drives *β*‐catenin accumulation in the cytoplasm, which then translocates to the nucleus, where it functions as a transcription co‐factor. This nuclear *β*‐catenin drives the expression of genes involved in epithelial to mesenchymal transition (EMT), invasion, and metastasis [51].

In GBC, CTNNB1 mutations occur at a frequency of nearly 5%–6% as reported by both TCGA and COSMIC datasets. Exon 3 serves as the mutational hotspot, with recurrent missense mutations including D32V/N/Y, S33F, S37F, T41A, and S45P/F. These mutations disrupt the phosphorylation‐dependent degradation of *β*‐catenin, resulting in its constitutive activation and oncogenic signalling [52]. Such dysregulation contributes to tumorigenesis and correlates with aggressive disease phenotypes.

Table 1 summarizes the most frequent mutation sites identified for each protein and their corresponding mutation frequencies as reported in GBC studies. This table provides an overview of mutational hotspots critical for understanding the molecular pathogenesis of GBC and highlights targets for precision therapeutic intervention. Frequencies were extracted from multiple cohorts and reflect the heterogeneity and prevalence of these genetic alterations across patient populations.

**TABLE 1:** The most frequent mutation site of each protein and their frequency reported in GBC.

Gene	Type	Mutation frequency (TCGA) (%)	Mutation frequency (COSMIC) (%)	Most frequent mutations	OncokB implication	Effect
TP53	Tumor suppressor	58.6	44	R248Q/W, R273H/C/L, R175H/C, Y234C/N/H, S241F/C/Y, G245S/D/V, H179D/R/L, R280T/K, E285K, R337H	Likely oncogenic/oncogenic	Likely loss of function/loss of function
SMAD4	Tumor suppressor	21.1	14	R361C/H/G/S, D493N, G386R/D/V, D351G/H/N/Y, E526Q	Likely oncogenic	Likely loss of function/Loss of function
PIK3CA	Oncogene	10.3	7	E545K/G, E542K, H1047R, Q75E, M1043I/V, E726K, E81K, R88Q, G118D, N345S/T	Likely oncogenic/Oncogenic	Likely Gain of function/Gain of function
CDKN2A	Tumor suppressor	8.7	10	D108N/Y, D84N, H83Y, P48R, P114L, Q50P	Likely oncogenic/Oncogenic	Likely loss of function/Loss of function
ARID1A	Tumor suppressor	20	16	N218T, Q487*, Q515*, Q581*	Likely oncogenic	Likely loss of function
ARID2	Tumor suppressor	7.7	8	S297P, E267*, Q819*	Likely oncogenic	Likely loss of function
KRAS	Oncogene	6.9	12	G12D/A/C/R, L19F, G13D, Q61H	Oncogenic	Gain of function
ELF3	Context dependent	10.7	—	F303L, K304Qfs*167	Likely oncogenic	Likely loss of function
ERBB3	Oncogene	6.6	6	V104L, G284R, M91I, T355I, E332K, D297Y, S846I	Likely oncogenic/Oncogenic	Likely gain of function/Gain of function
ERBB2	Oncogene	6.6	5	S310F/Y, R678Q, L755S, D769Y, V842I	Likely oncogenic/Oncogenic	Likely gain of function/Gain of function
STK11	Tumor suppressor	6.3	—	Q159*, S216F, G251R, G196R	Likely oncogenic	Likely loss of function
CTNNB1	Oncogene	5.5	5	S45P/F, D32V/N/Y, S37F, S33F, T41A	Likely oncogenic/Oncogenic	Likely Gain of function/Gain of function

## 3 Signalling Crosstalk of Frequently Mutated Proteins Found in Gall Bladder Cancer

Mutations in key regulatory proteins not only disrupt their intrinsic functions but also perturb a plethora of signaling pathways, leading to altered cellular phenotype. Some of these mutations confer gain‐of‐function properties that enhance oncogenic potential, manifesting as increased proliferation, migration, invasion, and resistance to conventional therapeutics [53].

Among these key regulatory proteins, the tumor suppressor p53 emerges as the most frequently mutated gene across many human cancers, including GBC, as shown in meta‐signature analyses of GBC [54]. The p53 protein plays a critical role in regulating cell growth, cell development, cell stress, apoptosis, DNA repair, and senescence. Mutations in TP53, particularly missense mutations within its DBD, not only lead to loss‐of‐function in terms of native functions but also the acquisition of numerous oncogenic traits. In addition, TP53 mutations have been associated with histopathological differentiation and clinicopathological features such as tumor grade, lymph node invasion, and TNM stage in GBC [55].

Mutant p53 exists in diverse cancers such as lung, breast, pancreas, oral, etc., where it promotes oncogenic traits by producing oncogenic miRNA biogenesis, transactivation of chromatin modulators and 20S/26S proteasome, resistance to cisplatin (CDDP), and sequestering other tumor suppressor proteins in p53 amyloid fibres [56, 57]. For instance, mutant p53R273H in colon adenocarcinoma and R175H in breast cancer downregulate four key miRNAs (miR‐412, miR‐455‐3p, miR‐519e, miR‐562), which in turn regulate key cellular processes such as apoptosis, cell cycle arrest, and EMT & migration repression. Similarly, mutant R273H in breast cancer hyperactivates histone acetyltransferases and methyltransferases, further driving tumor progression (Figure 3a). These oncogenic activities rendered by this mutant p53 are also associated with cisplatin resistance and recurrence in head and neck cancer. However, our literature search did not identify any studies investigating the p53R273H mutation in GBCs.

**FIGURE 3:**
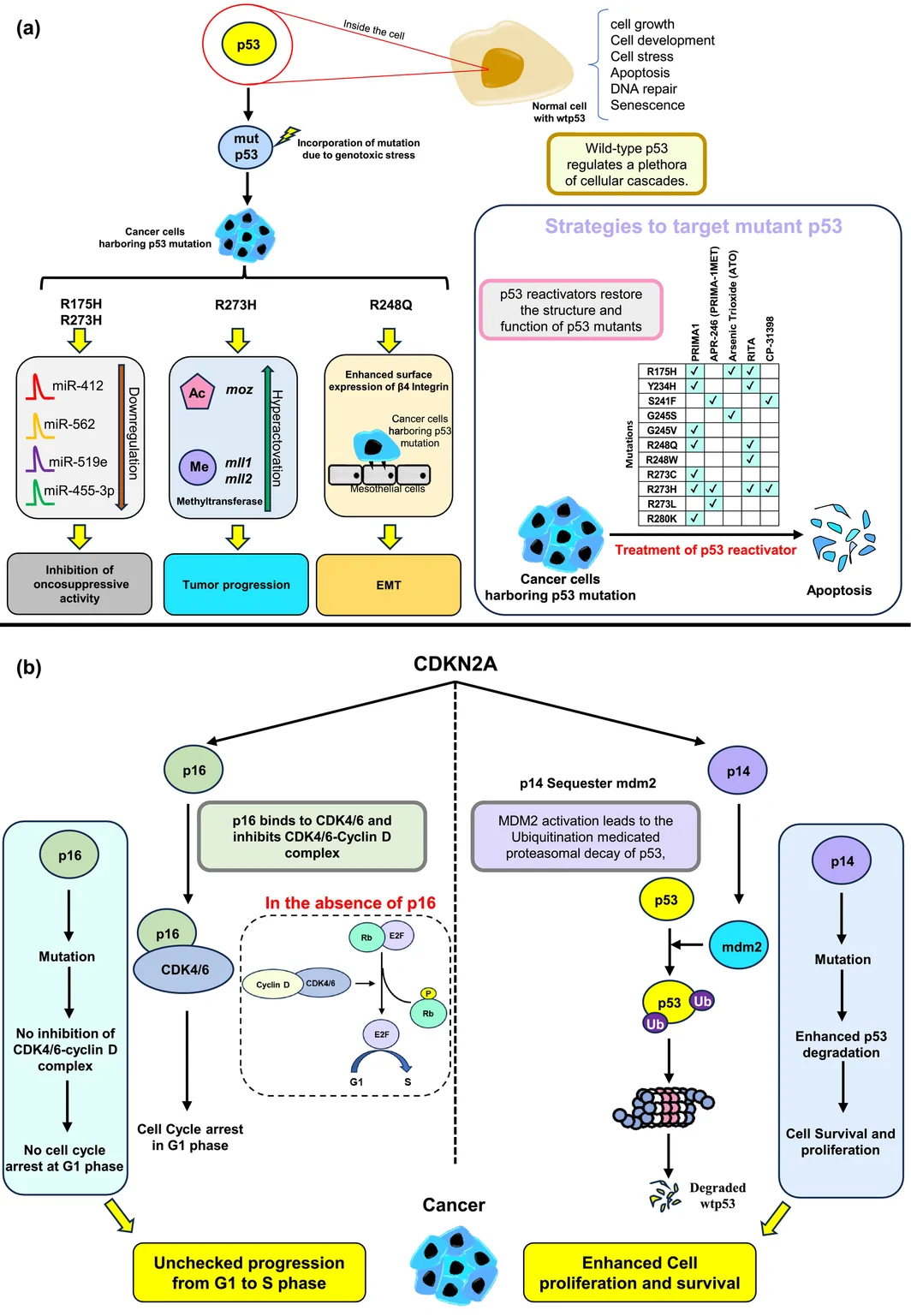
Impact of p53 and CDKN2A mutations in tumorogenesis. (a) The tumor suppressor protein p53, often referred to as the “guardian of the genome,” orchestrates a wide array of cellular regulatory cascades. Upon acquiring mutations, this master regulator becomes functionally compromised and may acquire oncogenic properties. Mutant p53 can exert gain‐of‐function effects by suppressing onco‐suppressive microRNAs, promoting tumor progression through hyperactivation of chromatin remodelers, methyltransferases, and acetyltransferases, and facilitating epithelial‐to‐mesenchymal transition (EMT) via upregulation of *β*4‐integrin expression. These oncogenic functions, however, can be counteracted by a variety of p53 reactivators that restore its tumor‐suppressive activity. (b) The CDKN2A gene encodes two distinct tumor suppressor proteins: P14/ARF and p16/INK4a. p14/ARF regulates p53 stability by inhibiting its negative regulator MDM2. Mutations in p14/ARF lead to increased MDM2 activity, resulting in enhanced p53 degradation and promoting cell survival and proliferation. p16/INK4a binds to CDK4/6, preventing their interaction with cyclin D and enforcing G1 phase cell cycle arrest. Mutations in p16/INK4a disrupt this checkpoint control, enabling uncontrolled cell cycle progression and contributing to tumorigenesis.

Another frequently mutated gene in GBC is CDKN2A, one of the regulators of p53. This gene encodes two critical tumor suppressor proteins: p14 and p16. Protein p14 (ARF) maintains p53 stability by sequestering its negative regulator, MDM2. Mutations in p14 disrupt this interaction, resulting in unchecked MDM2 activity and accelerated degradation of p53, ultimately culminating in enhanced cell proliferation and survival [58]. On the other hand, p16 (INK4A) binds to CDK4 and CDK6, preventing the formation of the cyclin D‐CDK4/6 kinase activity complex. This inhibition halts cell cycle progression at the G1 phase by inhibiting the phosphorylation of the Rb protein (Figure 3b). Hence, mutation(s) in CDKN2A impair this checkpoint control, allowing uncontrolled transition through the G1 phase and enhancing the proliferative capacity of cells [59]. CDKN2A alterations are strongly associated with increased susceptibility to a broad range of cancers, including GBC, where they have also been linked to better pathological tumor differentiation [60].

SMAD4, a central mediator of the TGF‐*β* signaling pathway, is frequently mutated in various cancers, with the mutation hotspot predominantly located in its MH2 domain and, to a lesser extent, in the MH1 domain. These mutations lead to the inactivation of the TGF‐*β* signaling through various mechanisms. For instance, mutations in the MH1 domain, such as R135*, impair the DNA‐binding capacity of SMAD4, thereby preventing the transcriptional activation of various tumor suppressor genes and blocking TGF‐*β*‐mediated growth inhibition [26]. In GBC, several deleterious missense mutations, including D351N, R361C, G352E, R361H, and E526Q, have been reported. These variants are thought to destabilize the SMAD4 protein and may contribute to GBC pathogenesis [61]. In a separate large‐scale analysis, Rimini et al. demonstrated SMAD4 mutations were associated with improved progression‐free survival (PFS) (hazard ratio [HR] = 0.49, *p* = 0.018) and overall survival (HR = 0.11, *p* = 0.023) in GBCs [62]. In colorectal cancer, mutations within the MH2 domain disrupt the formation of functional SMAD4 complexes by inhibiting the interaction between SMAD4 and phosphorylated SMAD2/3. This disruption results in uncontrolled cell proliferation, aiding tumorigenesis. A notable example is the R361C mutation (in colorectal cancer), located in the C‐terminal region of SMAD4, which impairs its oligomerization and compromises its pro‐apoptotic functions. Instead of promoting apoptosis, the mutated SMAD4 interacts aberrantly with Lef1, leading to activation of the Wnt/*β*‐catenin signaling pathway. This oncogenic shift enhances tumor progression and increases the likelihood of distant metastasis in affected individuals (Figure 4a) [63, 64].

**FIGURE 4:**
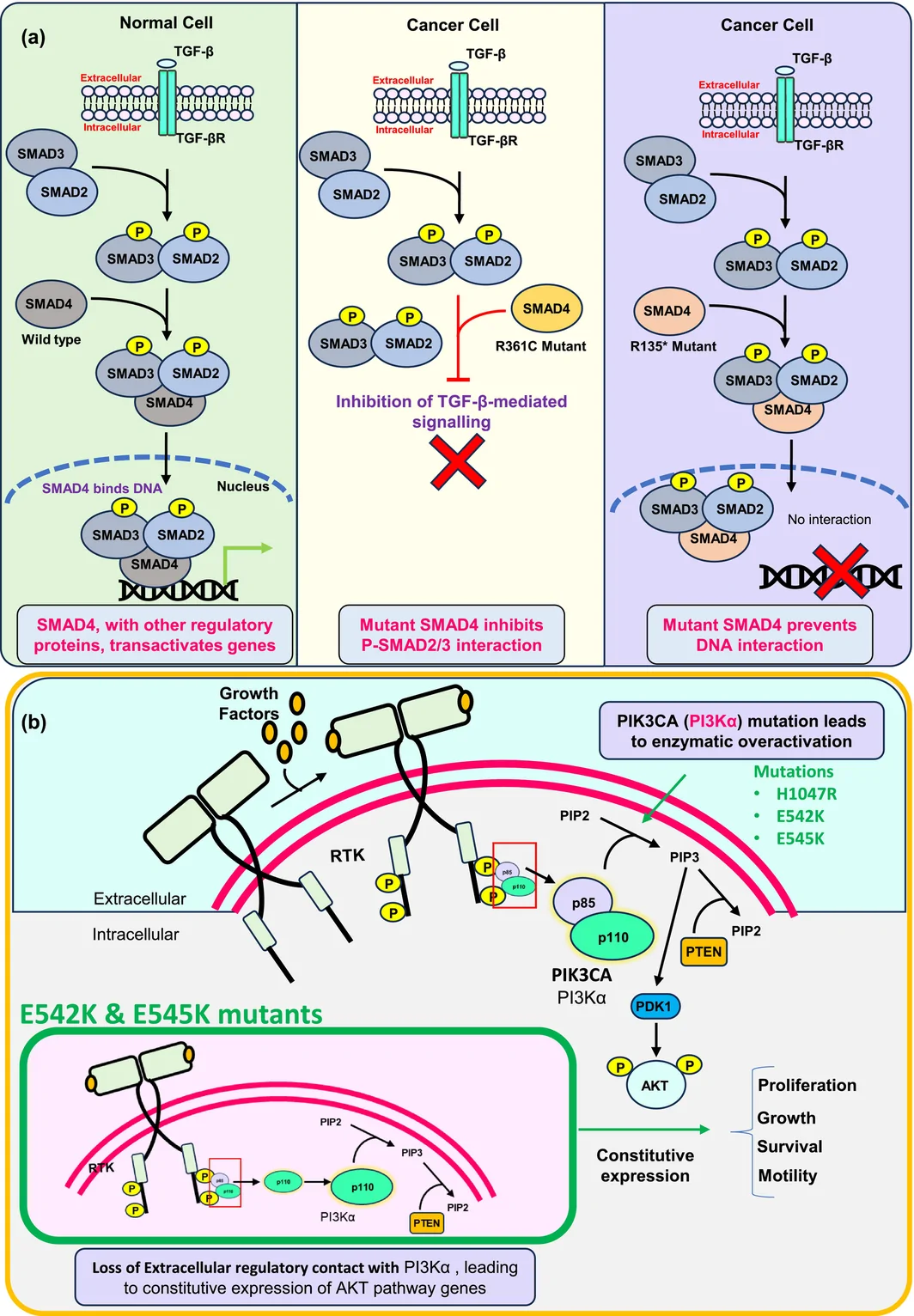
Oncogenic potential of mutated SMAD4 and PI3K*α* in human cancers. (a) In normal cells (left panel), TGF‐*β* signaling induces phosphorylation of SMAD2/3, which subsequently interacts with SMAD4 to form a transcriptionally active complex that translocates into the nucleus and activates target genes essential for cellular homeostasis. In cells harboring the SMAD4‐R361C mutation (middle panel), the mutant SMAD4 fails to interact with phosphorylated SMAD2/3, thereby disrupting canonical TGF‐*β* signaling. Conversely, in cells expressing the SMAD4‐R135* truncation mutant (right panel), the mutant protein retains the ability to form a complex with phosphorylated SMAD2/3 and translocate into the nucleus, but is unable to bind DNA, rendering the complex transcriptionally inactive. (b) Growth factor‐mediated activation of receptor tyrosine kinases (RTKs) stimulates both subunits of PI3K*α*—p85 (regulatory) and p110 (catalytic), which catalyze the conversion of phosphatidylinositol‐4,5‐bisphosphate (PIP2) to phosphatidylinositol‐3,4,5‐trisphosphate (PIP3). This lipid second messenger activates downstream AKT signaling, promoting cell proliferation, growth, survival, and metabolic regulation. In cancer cells harboring activating mutations in PI3K*α*, the extracellular regulatory control is lost, leading to constitutive AKT pathway activation and aberrant expression of pro‐survival and proliferative genes.

PIK3CA encodes the p110*α* catalytic subunit of phosphatidylinositol 3‐kinase alpha (PI3K*α*), a key heterodimeric lipid kinase comprising a p110 catalytic subunit and p85 regulatory subunit. PI3K*α* plays a central role in the PI3K/Akt signaling pathway, which governs key cellular processes such as proliferation, growth, survival, and motility. Structurally, PI3K*α* is composed of several domains—including the kinase, helical, C2, Ras‐binding, and N‐terminal (p85‐binding) domains—that coordinate the phosphorylation of phosphatidylinositol‐4,5‐bisphosphate (PIP2) at the 3′ hydroxyl position, generating phosphatidylinositol‐3,4,5‐triphosphate (PIP3) [29, 65].

PIK3CA mutations, especially in the kinase (H1047R) and helical domains (E542K, E545K), are among the most frequent genetic alterations in human cancers and drive enzymatic overactivation, promoting tumor development [66]. Helical domain mutants of PIK3CA gain activity by disrupting the inhibitory interaction of the helical domain of p110*α* and regulatory subunit p85, hence catalyse the conversion of PIP2 to PIP3 regardless of extracellular signals, raising intracellular PIP3 levels and continuously recruiting AKT to the membrane. Augmented phosphorylation of AKT and mTOR renders cells resistant to apoptosis, supports metabolic reprogramming, enhances motility, and induces resistance to anti‐cancer therapies [67]. Kinase mutation H1047R mechanistically increases PI3K*α* activity by inducing structural changes in the kinase domain, which abolish the auto‐inhibitory effect of the C‐terminal tail, enhance the protein's positive charge at membrane‐binding regions, and increase membrane affinity. This mutation substitutes the RAS dependent activation of PIK3CA and converts PI3K*α* into a hyperactive state, with increased basal and membrane‐stimulated lipid kinase activity (Figure 4b) [68]. Moreover, the phenotypic impact of these mutants on cancer development can occur through different mechanisms in different cancers. In GBC, the E545K mutation is the most frequent PIK3CA alteration and has been associated with patient survival. In vitro studies have shown that this mutation reduces proliferation of GBC cells, and in vivo studies have demonstrated diminished tumor growth [55]. Other recurrent PIK3CA mutations reported in GBC include E542K, E545G, H1047L, and H1047R [69]. Further, in cervical cancer, mutant E542K and E545K are reported to promote proliferation and glycolysis by inducing *β*‐catenin/SIRT3 signaling pathway [70]. In breast cancer, mutant PIK3CA aids in cell proliferation, suppression of apoptosis and chemotherapy resistance by activating PI3K/AKT/mTOR pathway and has been associated with poor prognosis [32, 71].

ARID1A and ARID2 (the AT‐rich interactive domain) proteins are two important subunits of the tumor‐suppressing chromatin remodelling complex Mammalian SWItch/Sucrose Non‐Fermenting (SWI/SNF), wherein they aid in making chromatin more accessible for the transcription of genes regulating key cellular processes such as damage repair, proliferation and genomic stability [72]. Mutated ARID1A destabilized the complex and impairs chromatin remodelling, causing dysregulation of gene regulation. It is also reported to have caused dysregulation of the cell cycle checkpoint of G0–G1, leading to uncontrolled tumor growth of various ovarian, breast and liver cancer cell lines. It activates oncogenesis by triggering the PI3K/AKT/mTOR pathway and synergizing with mutations of other oncogenes such as PIK3CA [73]. ARID1A also influences the expression of EMT‐related genes by binding to their promoters; hence, while ARID1A is mutated, it is unable to bind the promoters and lead to induction of their expression. For instance, in gastric cancer cell lines, ARID1A silencing results in the expression of N‐cadherin and vimentin, whereas in neuroblastoma it leads to the induction of matrix‐metalloproteinase‐2 (MMP‐2) and MMP‐9 expression with a decrease in E‐cadherin expression. Mutated ARID1A also impairs the DNA mismatch repair pathway and is associated with high microsatellite instability in gastric, colorectal and uterine endometrioid carcinoma (Figure 5a) [74]. Mutated ARID1A is associated with poor overall survival and disease progression in different cancers, including GBC. In support, Nan et al. demonstrated that loss of ARID1A expression in GBC leads to PD‐L1 overexpression and impaired tumor‐infiltrating lymphocyte function, thereby promoting immune evasion and contributing to poor patient prognosis [38]. In a recent study, patients with ARID1A‐deficient biliary tumors exhibited significantly shorter overall survival compared to those with ARID1A‐proficient tumors, as defined by immunohistochemical staining (median OS: 12 vs. 30 months) [75]. On similar lines, mutations in the C2H2 zinc finger domain of ARID2 prevent it from recruiting DNMT1 at the promoters of EMT‐related genes such as SNAIL, leading to metastasis in hepatocellular carcinoma (Figure 5a) [76]. ARID2 mutation is also associated with upregulation of cyclin D1, cyclin E1, CDK4 and enhanced phosphorylation of the Rb protein in hepatocellular carcinoma, thereby augmenting tumorigenesis [77]. Collectively, mutations in ARID2 correlate with worse clinical outcomes and increased metastatic potential in various cancers, including hepatocellular carcinoma, renal cell carcinoma and melanoma [76].

**FIGURE 5:**
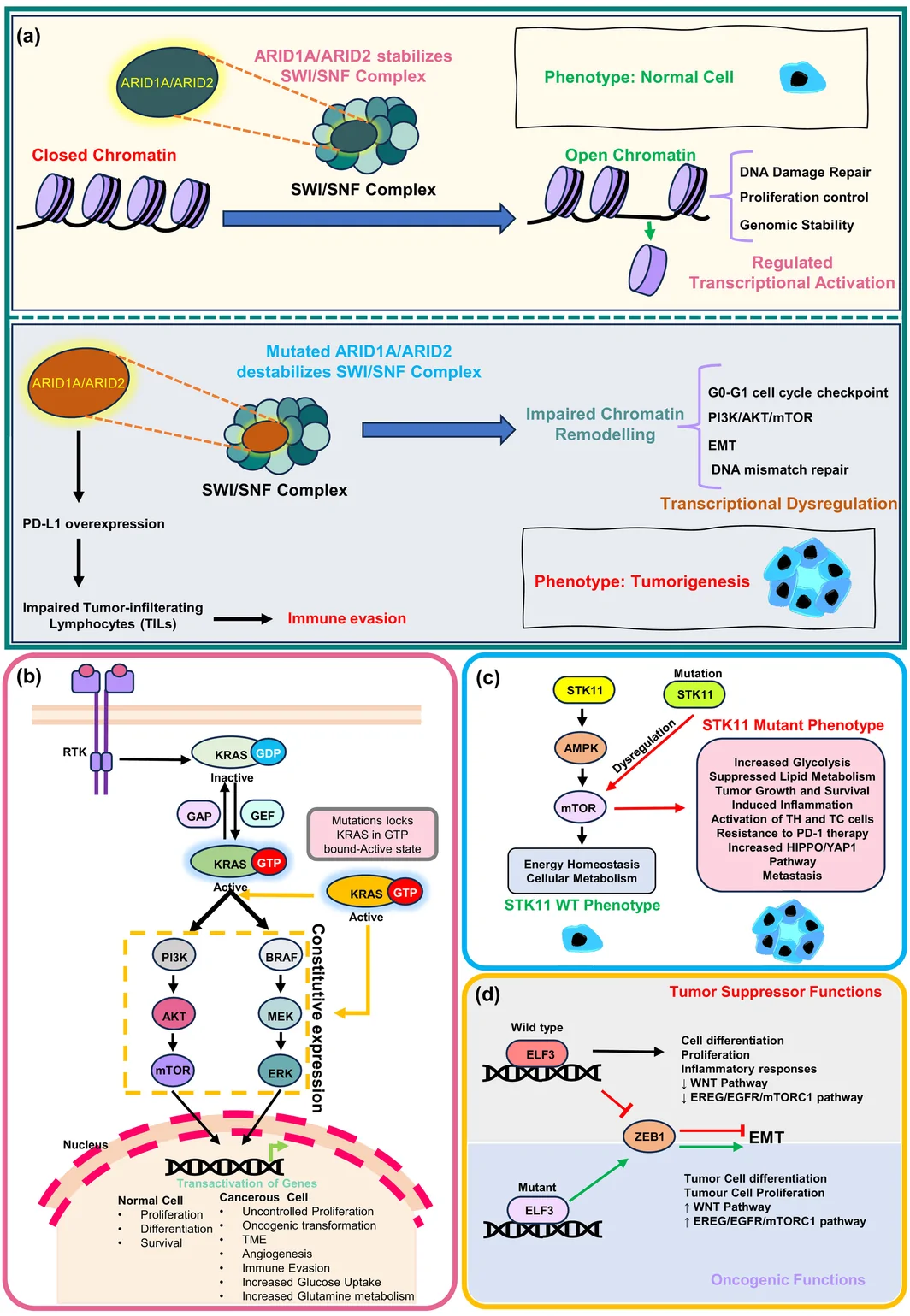
Oncogenic roles of ARID1A/ARID2, KRAS, STK11 and ELF3 mutations in human cancers. (a) ARID1A and ARID2 are integral components of the SWI/SNF chromatin remodeling complex, which facilitates the transition from closed to open chromatin states, thereby enabling transcriptional activation of genes involved in DNA damage repair, proliferation control, and genomic stability. Mutations in ARID1A/ARID2 destabilize the SWI/SNF complex, impairing chromatin remodeling and leading to dysregulation of DNA mismatch repair, epithelial–mesenchymal transition (EMT), PI3K/mTOR signaling, and G0, G1 cell cycle arrest. (b) Receptor tyrosine kinase (RTK) activation promotes GDP‐to‐GTP exchange on KRAS, triggering downstream signaling through mTOR and ERK pathways that support normal cell survival and proliferation. Oncogenic mutations in KRAS lock the protein in its GTP‐bound active state, resulting in constitutive activation of proliferative and survival pathways that drive malignant transformation. (c) STK11 (also known as LKB1) plays a pivotal role in maintaining energy homeostasis and regulating cellular metabolism through modulation of the mTOR signaling pathway. Loss‐of‐function mutations in STK11 disrupt this regulatory axis, leading to aberrant mTOR activation and promoting oncogenic phenotypes characterized by uncontrolled growth and metabolic reprogramming. (d) The tumor suppressor ELF3 transforms into an oncogene upon mutation.

KRAS is a small GTPase protein that acts as a molecular switch regulating key cellular signaling pathways such as MAPK and PI3K/AKT, which control cell proliferation, differentiation, and survival. Normally, KRAS cycles between an active GTP‐bound state and an inactive GDP‐bound state, transmitting external growth signals to the nucleus to regulate these processes [78]. Mutations in KRAS—most frequently at codons 12, 13, and 61—impair its intrinsic GTPase activity, locking KRAS in a constitutively active GTP‐bound form. This continuous activation leads to persistent downstream signaling pathways such as PI3K/AKT and MAPK/ERK, independent of growth factors, driving uncontrolled cell growth and oncogenic transformation [79]. Mutant KRAS also upregulates production of inflammatory cytokines like IL‐6, IL‐8, and chemokines that recruit immune and stromal cells. This creates a pro‐tumorigenic TME that supports cancer growth, angiogenesis, and immune evasion [80]. KRAS mutation also aids in induction of expression of PD‐L1 on tumor cells due to the stabilization of its mRNA and downregulates MHC‐I molecules, impairing cytotoxic T cell recognition and enabling immune escape [81]. KRAS mutations are also implicated in metabolic reprogramming of colorectal cancer cells wherein these induce increased glucose uptake and glutamine metabolism to supplement the higher energy demands of rapidly proliferating tumor cells. Also, KRAS mutations are reported to promote angiogenesis in colorectal cancer (Figure 5b) [82]. Regional studies from northern and north‐eastern Indian states have reported a higher frequency of codon 12 mutations in GBC tissues. These mutations have been associated with several clinical features, including cellular differentiation, tumor grade, and TNM stage, as well as markedly poorer overall survival [83, 84]. In a separate study, Gelfer et al. showed across both resectable and unresectable cohorts, KRAS G12 mutations were associated with significantly poorer overall survival in intrahepatic cholangiocarcinoma, but not in extrahepatic cholangiocarcinoma or gallbladder adenocarcinoma, when compared with wild‐type KRAS or other KRAS variants [85]. These oncogenic traits of KRAS mutants result in development of resistance to chemotherapy and are associated with poor prognosis, higher relapse rates, shorter overall survival and disease‐free survival in multiple cancers [41].

STK11, a serine/threonine kinase acts as a tumor suppressor by regulating energy homeostasis, and cellular metabolism by phosphorylating kinase domain of AMPK and other AMPK related kinases, thereby activating AMPK signaling. It restricts the growth of cells in energetically unfavorable conditions, thus maintaining normal metabolism and cellular polarity [50]. Mutations in STK11 disrupt its tumor suppressive functions, leading to unchecked mTOR signaling, increased glycolysis, suppressed lipid metabolism, and a cellular metabolic shift conducive to rapid tumor growth and survival, as frequently observed in cancers such as non‐small cell lung cancer, ovarian cancer, breast cancer, and colorectal cancer. In lung adenocarcinoma, STK11 mutations are reported to reduce infiltration and activation of CD4+ and CD8+ T cells, along with decreased interferon signaling [86]. Also, STK11 mutant non‐small cell lung carcinoma tumors are found to exhibit lower PD‐L1 expression, yielding an immunologically cold tumor microenvironment and developing resistance towards anti‐PD‐1 therapy [87]. Not only this, mutation in STK11 has been directly linked with upregulation of the HIPPO/YAP1 pathway, leading to increased YAP1‐mediated transcription of cytokines (IL‐6, CXCL8, CXCL2) and extracellular matrix proteins in lung adenocarcinoma. These events, in turn, support aggressive tumor growth and metastasis and negatively influence patient prognosis and therapeutic outcomes in various cancers (Figure 5c) [88]. Evidence on STK11 mutations in gallbladder carcinoma remains limited, and their functional impact on disease progression warrants further investigation in future studies.

ELF3 (E74 Like ETS Transcription Factor 3), a member of the ETS family of transcription factors predominantly expressed in epithelial tissues, plays a crucial role in regulating gene expression programs involved in epithelial cell differentiation, proliferation, and inflammatory responses [89]. Being a transcription factor, it regulates a wide range of genes involved in differentiation and proliferation of cells. normally, it negatively regulates the expression of genes involved in WNT pathway. It also represses the expression of the EMT transcription factor ZEB1, thereby inhibiting the EMT phenotype. Mutated ELF3 fails to execute this regulation, which leads to increased EMT and the oncogenic phenotype of cells [90]. In GBC, it acts as a tumor suppressor by downregulating the EREG/EGFR/mTORC1 signaling pathway. Loss‐of‐function mutations in ELF3 lead to upregulation of epiregulin (EREG), which activates EGFR and mTORC1, promoting tumor cell proliferation, epithelial‐mesenchymal transition (EMT), and invasion (Figure 5d) [46]. These phenotypic effects of ELF3 mutations correlate with aggressive features and poor prognosis in cancers including lung adenocarcinoma, colorectal cancer, GBC, and ovarian cancer [91]. In a recent study utilizing GBC tissues, reduced ELF3 expression was shown to correlate with advanced clinical stage and deeper tumor invasion. Moreover, allograft mouse models derived from KPCE (Trp53^R172H^; Elf3^f/f^) and KPC (Kras^G12D^; Trp53^R172H^; Elf3^wt/wt^) organoids revealed that KPCE allograft tumors developed poorly differentiated structures characterized by mTORC1 activation and a mesenchymal phenotype [46].

ERBB2 (HER2) and ERBB3 (HER3) are members of the epidermal growth factor receptor (EGFR/ERBB) family of receptor tyrosine kinases, each playing key roles in cellular growth, differentiation, and survival signaling [92]. ERBB2/HER2 lacks a direct ligand‐binding site and instead forms heterodimers with other EGFR family members such as EGFR or HER3 to mediate signaling. In normal cells, HER2 is expressed at low levels, tightly regulated by complex formation with chaperones like HSP90 (Figure 6a). In cancer, mutated ERBB2 leads to persistent overexpression and stable homodimer formation, resulting in ligand‐independent activation of downstream proliferative pathways including MAPK and PI3K/AKT. This unchecked signaling drives aggressive tumor growth and is commonly implicated in breast, lung, and several other cancers (Figure 6b) [93]. On the other hand, ERBB3/HER3 has an impaired kinase domain, hence lacks significant intrinsic catalytic activity. It becomes functionally important through strong heterodimerization with HER2 and activation of PI3K and MAPK signaling cascades. In healthy tissue, ERBB3 participates in tightly controlled growth and differentiation responses (Figure 6a) [94]. Extracellular domain mutations like S310F/Y induce strong receptor dimerization by introducing hydrophobic aromatic side chains, promoting ligand‐independent activation and kinase activity with enhanced interaction with EGFR and HER3 [95]. In breast cancer, mutation L755S in the kinase domain of ERBB2 protein has been shown to confer strong transformation activity and resistance to targeted therapy [96]. On similar lines, other mutations in the kinase domain of the protein such as V842I, D769Y/H demonstrated constitutive kinase activity, driving persistent downstream signaling such as the PI3K/AKT pathway, as observed in breast, lung, and endometrioid cancers [93, 97]. Owing to the molecular effects of these mutations, ERBB2 mutant cancer cells exhibit robust transformation, aggressive, and therapy‐resistant phenotype. Zhu et al. reported that ERBB2 expression was markedly elevated through the synergistic interaction between GBC and pancreaticobiliary malfunction [98]. In a recent study employing whole‐exome sequencing, Gao et al. identified the ERBB2 I655V mutation as a potential determinant of therapeutic response in GBC, noting that this variant constitutively activates ERBB2 and drives sustained PI3K–AKT and MAPK–ERK pathway signaling [99]. Cancer‐associated ERBB3 mutations enhance dimerization or interaction with HER2, leading to abnormal activation of downstream survival and growth signals [94]. Mutant‐driven overexpression of ERBB3 contributed to reduced T cell infiltration, leading to immunosuppression in the tumor microenvironment and therapy resistance in breast, cervical, and GBC (Figure 6b) [100]. No substantive studies have yet examined ERBB3 mutations in gallbladder carcinoma, highlighting an important gap that warrants exploration in future research.

**FIGURE 6:**
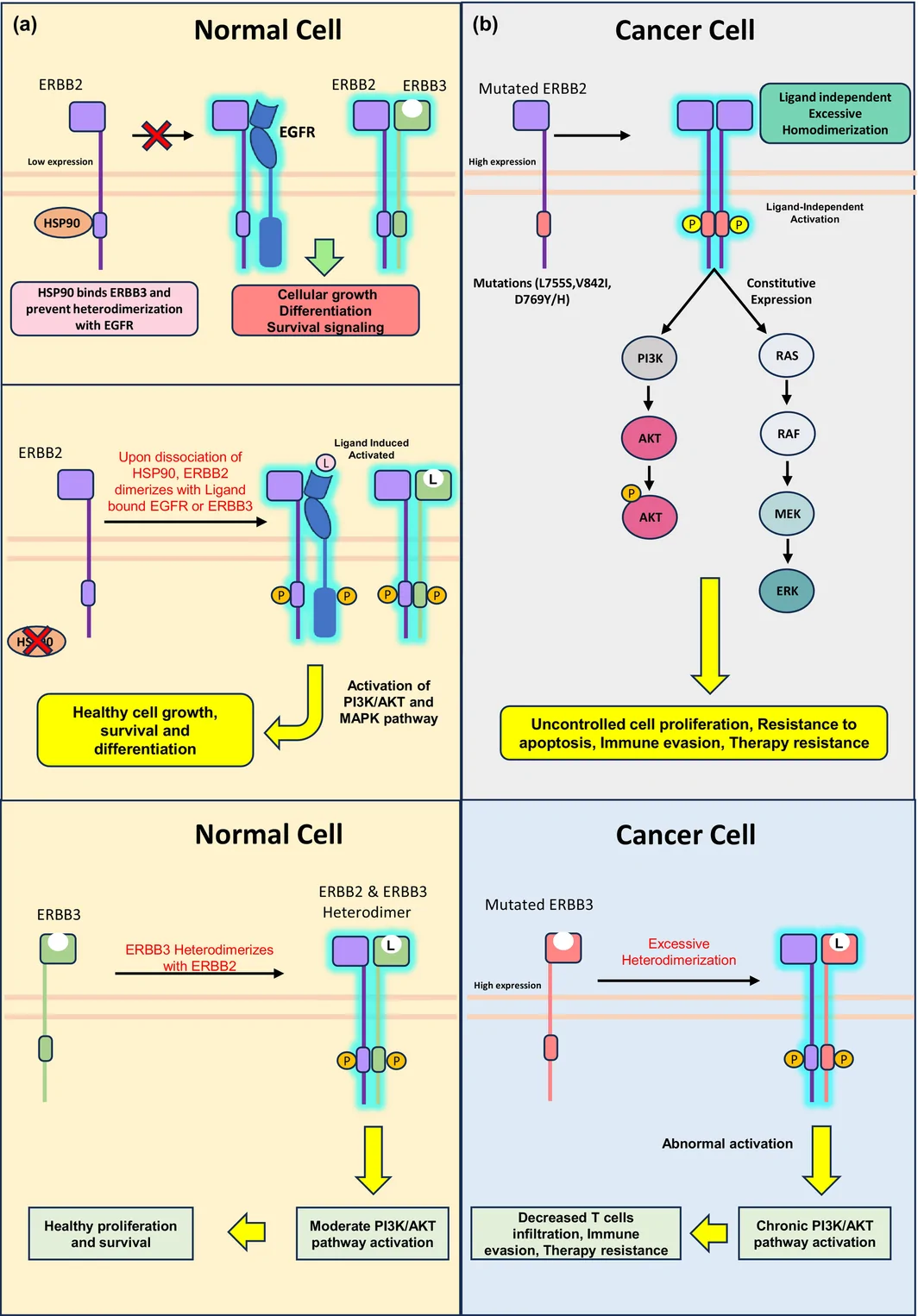
Functional roles of normal and mutated ERBB2 and ERBB3. (a) Left panel (normal cells): In the absence of ligand, HSP90 binds to ERBB2 and inhibits its dimerization with EGFR or ERBB3, thereby maintaining tight regulatory control. Upon ligand stimulation, HSP90 dissociates from ERBB2, allowing it to heterodimerize with EGFR or ERBB3. This interaction leads to phosphorylation of the cytoplasmic tails, triggering downstream activation of the PI3K/AKT and MAPK signaling pathways. ERBB3, upon heterodimerization with ERBB2, specifically mediates PI3K/AKT signaling, supporting normal cell proliferation, differentiation, and survival. (b) Right panel (mutated cells): Mutations in ERBB2 disrupt HSP90‐mediated regulation and confer homodimerization capability, resulting in constitutive activation of PI3K/AKT and MAPK pathways, thereby promoting oncogenesis and metastasis. Similarly, mutations in ERBB3 enhance its heterodimerization with ERBB2, leading to hyperactivation of PI3K/AKT signaling and favoring malignant transformation.

CTNNB1 encodes beta‐catenin, a multifunctional protein with pivotal roles in both cell–cell adhesion and gene regulation. *β*‐catenin anchors cadherin‐based adherens junctions at the cell membrane, thereby maintaining tissue integrity, while also serving as a core effector of the Wnt signaling pathway and acting as a transcriptional co‐activator for Wnt‐responsive genes critical to cell fate, proliferation, and differentiation [101]. Mutations in serine/threonine/residues of exon 3 of CTNNB1 such as S45P/F, S37F, S33F, and T41A, that are normally phosphorylated by GSK3*β* and casein kinase 1 to mark it for ubiquitin‐mediated proteasomal degradation, drive cancer development by preventing the degradation of *β*‐catenin, causing its accumulation in nucleus and cytoplasm, which further constitutively activates WNT target genes, promoting cancer phenotypes including stemness, survival, and proliferation (Figure 7) [51]. Mutations also affect the ability of *β*‐catenin to anchor the adherens junctions that further facilitate epithelial to mesenchymal phenotype. Nuclear accumulation of *β*‐catenin is correlated with poor prognosis in breast and colorectal cancer [102, 103].

**FIGURE 7:**
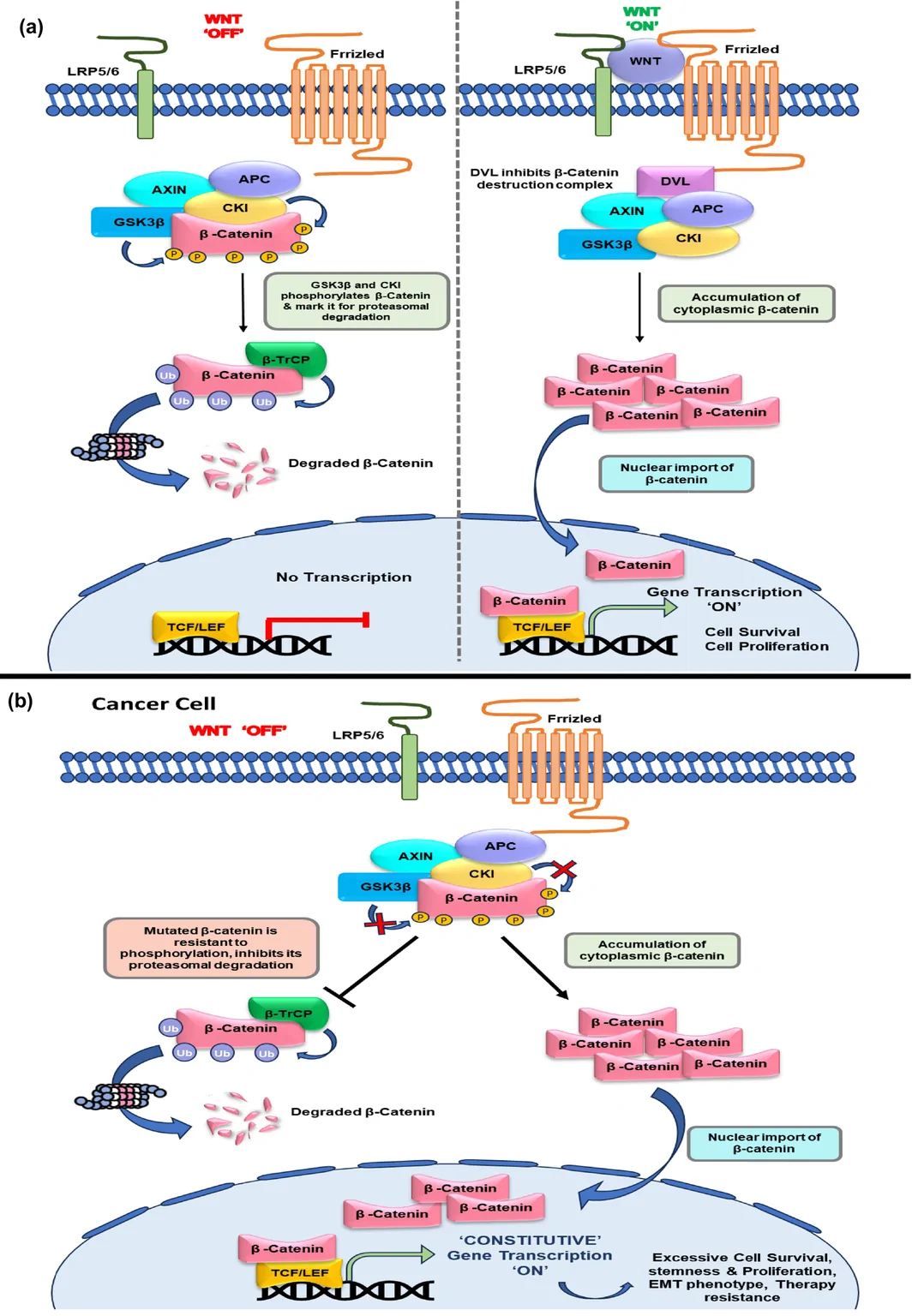
Involvement of wild‐type and mutated *β*‐catenin signalling in Wnt signalling. (a) Wild‐type *β*‐catenin: In the absence of Wnt ligand, the *β*‐catenin destruction complex (comprising GSK3*β* and CKI kinases) phosphorylates *β*‐catenin, marking it for proteasomal degradation. This prevents its nuclear translocation and subsequent activation of target gene transcription. Upon Wnt stimulation, Dishevelled (DVL) inhibits the destruction complex, allowing *β*‐catenin to accumulate in the cytoplasm and translocate into the nucleus, where it activates transcription of Wnt‐responsive genes involved in cell proliferation and differentiation. (b) Mutated *β*‐catenin: Mutations in *β*‐catenin impair its phosphorylation and degradation by the destruction complex, even in the absence of Wnt signaling. This results in constitutive nuclear accumulation and transcriptional activation of target genes, promoting uncontrolled cell proliferation and tumorigenesis.

Beyond the well‐established mutations already discussed in this review, several additional genomic alterations may represent future therapeutic vulnerabilities in GBC, even though many remain incompletely characterized or not yet functionally validated. Importantly, the recurrent mutations already highlighted in this review remain the most biologically and clinically relevant because they are linked to altered signaling, clinicopathological aggressiveness, and putative therapeutic response. Together, these mutational and signaling abnormalities underscore the possibility that GBC harbors additional yet‐unexploited targets that could be leveraged for biomarker‐driven diagnosis, prognosis, and precision therapy in the future.

## 4 Putative Treatment Strategies to Counter GBC

Management of GBC involves a range of conventional modalities, including surgery, chemotherapy, and radiotherapy, alongside emerging approaches such as targeted therapy and immunotherapy. Surgical resection remains the primary treatment for early‐stage disease and is considered the most effective option. In contrast, for advanced unresectable cases, chemotherapy or radiotherapy is incorporated into the therapeutic regimen. Radiation therapy employs high‐energy rays to eradicate malignant cells or suppress their proliferation, whereas chemotherapy relies on cytotoxic drugs to achieve the same therapeutic objective [104]. Currently, GC (Gemcitabine and cisplatin) regimen is the go‐to chemotherapy treatment plan for GBC. If chemotherapy is to be given with radiation therapy, 5‐fluorouracil or capecitabine are used for treatment [7]. Due to their non‐specific nature, these affect normal cells too, resulting in significant systemic toxicity. Also, resistance acquired by the cells to the chemotherapy drugs reduces their efficacy. All these factors collectively affect the survival of patients [105]. To overcome these issues, targeted therapy and immunotherapy are being considered from a treatment point of view. Immunotherapy leverages inhibitors targeting the interaction of specific proteins expressed on T cells and their complementary ligand expressed on cancer cells. Interaction of both protein and ligand prevents T‐cells from killing the cancer cells. For instance, pembrolizumab, nivolumab and Durvalumab target the interaction of checkpoint protein PD‐1 expressed on T cells and PDL1 ligand expressed on cancer cells. Similarly, ipilimumab prevents interaction of CTLA‐4 expressed on T cells and CD80/86 expressed on antigen presenting cells. Hence, these immune checkpoint inhibitors prevent immune cell evasion and keep immune system active. These drugs are often administered in combination with chemotherapy drugs. Phase 3 trials such as TOPAZ‐1 and KEYNOTE‐966 have established chemo‐immunotherapy as the first‐line treatment approach for GBC and other biliary tract cancers. These studies showed that adding durvalumab or pembrolizumab to the gemcitabine–cisplatin regimen improves overall survival in biliary tract cancer [106]. However, low expression of PD1 or CTL4A is the major limitation of immunotherapy, resulting in exclusion of majority of patients from this regime [107]. Furthermore, advanced sequencing technologies such as next generation sequencing (NGS) and RNA sequencing has led to the identification and characterization of novel potential therapeutic targets of GBC [105]. Targeted therapies rely on drugs that specifically identify and attack cancer cells based on some unique features of cancer cells such as biomarkers, gene mutations etc. SAFIR ABC‐10 is one such Phase 3 precision oncology clinical trial testing matched targeted maintenance therapy in advanced biliary tract cancers, including GBC, however results are pending (https://clinicaltrials.gov/study/NCT05615818). It aims to establish targeted agents as second line or maintenance strategies.

Table 2 summarizes drugs that target the most frequently mutated proteins in GBC, which are currently approved or under investigation for other cancer types. These agents hold potential for repurposing in GBC therapy based on shared molecular alterations.

**TABLE 2:** Drugs targeting common mutated proteins in GBC, approved or investigated in other cancers, with potential for repurposing.

Gene	Drug	Target	Mechanism	Model of study	References
TP53	PRIMA‐1	R175H, R273C/H, Y234H, G245V, R248Q, R280K	Restores mutant p53 and stabilize it to wtp53 conformation	Osteosarcoma cell line	[108, 109]
	APR‐246 (PRIMA‐1^MET^)	R273H/L, S241F	Restores mutant p53 and stabilize it to wtp53 conformation	Osteosarcoma, small cell lung cancer, colon cancer cell line	
	Arsenic Trioxide (ATO)	R175H, G245S	Restores mutant p53 and stabilize it to wtp53 conformation	NCI‐60 cell lines, in vivo mouse models	
	RITA	R175H, Y234H, R248W/Q, R273H	Restores transcriptional activity of mutant p53	Female SCID mice based in vivo study	[110]
	CP‐31398	S241F, R273H	Restores mutant p53 and stabilize it to wtp53 conformation	Osteosarcoma, colon cancer, ovarian cancer, breast cancer, lung cancer cell lines	[111]
SMAD4	TGF Beta inhibitors such as Galunisertib, Vactosertib	Pan‐mutant	Inhibits TGF‐*β* receptor I kinase	Pancreatic cancer	[112]
	Ro‐31‐8220 (bisindolylmalei‐mide derivative)	R361H	Restores mutant smad4 activity to wild type	Colon cancer cell lines	[113]
PIK3CA	Capivasertib+ Fulvestrant	R88Q, E542K, E545K/G, M1043I/V, H1047R	Inhibits mutant PIK3CA	In Breast cancer patients with Fulvestrant drug	[114]
	Alpelisib	E545K, H1047R	Inhibits mutant PIK3CA	In Breast cancer patients with Fulvestrant drug	[115]
	RLY‐2608	E542K, H1047R	Inhibits mutant PIK3CA	In Breast cancer patients	[116]
KRAS	Sotorasib, Adagrasib, Divarasib, Olomorasib, Garsorasib	G12C	Lock KRAS in inactive state	NSCLC, CRC, Solid tumors	[117]
	RMC‐9805, MRTX1133, HRS‐4642	G12D	Prevents activation of downstream signalling of KRAS	Pancreatic cancer	
	Monobody 12VC1‐derived PROTACs	G12V/C	Blocks effector binding		[118]
CDKN2A	Abemaciclib, Palbociclib, Ribociclib	Pan‐mutant	Inhibits CDK4/6, make CDKN2A mutants more sensitive		[119]
ARID1A	Tazemetostat	Pan‐mutant	Inhibits EZH2 inhibitor methyltransferase activity	Ovarian, gastric cancer cell lines	[120, 121]
	PLX2853	Pan‐mutant	Inhibits bromodomain containing protein 4 (BRD4)	Cancer cell lines, PDX models, gynaecologic cancer patients	[122, 123]
ERBB2	Monoclonal antibodies such as trastuzumab, Pertuzumab, Margetuximab etc. alone or with chemotherapy	Pan‐mutant	Targets Her2 receptor	Breast cancer, NSCLC patients	[124]
	Antibody drug conjugate such as ado‐trastuzumab emtansine (T‐DM1)	Pan‐mutant	ERBB2‐targeted antibody and microtubule inhibitory conjugate	NSCLC patients	
	Tyrosine Kinase inhibitors such as neratinib, lapatinib, or afatinib	Pan‐Mutant	Targets EGFR and ERBB2	Lung and breast cancer	
ERBB3	Monoclonal antibodies such as Patritumab, Seribantumab, Lumretuzumab, Elgemtumab	Pan‐mutant	Block ERBB3 activity, triggers degradation of ERBB3, and induce antibody dependent cell mediated cytotoxicity (ADCC)	Breast cancer, NSCLC, HNSCC, and other solid tumors	[125]
	Bispecific monoclonal antibodies such as Duligotuzumab (EGFR/ERBB3), MM‐111 (EGFR/ERBB3), Isitarumab (IGF1R/ERBB3), Zenocutuzuma (ERBB2/ERBB3)	Pan‐mutant	Inhibits EGFR and ERBB3 together for synergistic effect	Breast cancer, NSCLC, HNSCC, and other solid tumors	
	Antibody drug conjugates such as Patritumab deruxtecan		Inhibit ERBB3 and topoisomerase I	Breast cancer, NSCLC and other solid tumors	
STK11	Bemcentinib in combination with anti PD‐1 or pembrolizumab	Pan‐mutant	Inhibits tyrosine kinase receptor AXL	NSCLC	[126]

## 5 Conclusion and Future Directions

Environmental and lifestyle factors (such as chronic gallstone disease, infections, and dietary influences etc.) are known contributors of GBC pathogenesis. However, accumulating evidence indicates that molecular alterations are major drivers of its development and progression. This review synthesizes the mutational landscape of GBC within a broader pathway‐level framework, highlighting potential phenotypic consequences and therapeutic opportunities for personalized treatment approaches.

Recent genomic and transcriptomic studies have revealed that GBC is characterized not only by recurrent mutations in key cancer‐related genes but also by widespread disruption of genome‐maintenance pathways, including homologous recombination, mismatch repair, nucleotide excision repair, DNA replication, and cell‐cycle regulation [127]. Integrative systems‐biology studies have further supported this view, identifying molecular signatures, hub genes, and regulatory networks associated with GBC pathogenesis [128]. In addition, a proteogenomic study of GBC identified TP53 and ELF3 as key driver mutations that play pivotal roles in reshaping cellular signaling pathways [129]. Studies from Indian cohorts have consistently identified recurrent mutations and pathway‐level disruption in DNA repair, replication, and cell‐cycle networks, with hub genes including XAB2, XPA, RPA1, RAD51B, BRCA2, ATR, CCNB2, and RANBP2 [130]. These findings suggest that the transition from gallstone disease to malignancy involves coordinated disturbance of protein‐interaction and regulatory networks rather than isolated point mutations alone. These recurrent genomic alterations may function as clinically useful biomarkers in GBC. Diagnostic biomarkers can support non‐invasive detection of malignancy through liquid biopsy platforms, including ctDNA and bile‐derived cfDNA, particularly when tissue sampling is limited and entails invasive procedures in metastatic disease like GBC [131]. Unlike single‐site tissue sampling, liquid biopsy can capture spatial and temporal tumor heterogeneity, making it especially useful for identifying recurrent mutations such as TP53 and KRAS, as well as actionable alterations that may guide targeted treatment selection. In biliary tract cancers, cfDNA‐based profiling has successfully detected important aberrations including KRAS and TP53, and emerging studies suggest that integrated liquid biopsy approaches may further improve the detection of GBC and distinguish it from benign gallbladder disease [132]. Actionable genomic alterations in gallbladder and biliary tract cancers are not merely descriptive biomarkers but clinically meaningful determinants of prognosis and treatment response. Solangi et al. explored the relationship between actionable genomic alterations and patient survival. They showed that alterations in genes such as KRAS, ERBB2, and PIK3CA, as well as HRD signatures (HRDsig), have important prognostic and therapeutic implications. In particular, patients who received matched targeted therapies, including PARP inhibitors for HRD‐positive tumors and ERBB2‐directed therapies, experienced better survival outcomes than those treated with conventional chemotherapy [133]. HRDsig analysis shows that a distinct subset of tumors with homologous recombination deficiency may be identifiable beyond conventional HRR gene mutation testing and may therefore be relevant for PARP inhibitor selection [134]. Together, these mutations and biomarkers underscore the transition of GBC management from descriptive pathology toward biomarker‐driven precision medicine.

Additionally, one cannot exclude the aspect to exploit the gut–biliary microbiome to influence mutagenesis and treatment response. Precision antibiotics, next‐generation probiotics/synbiotics, and fecal microbiota transplantation could restore eubiosis, lower genotoxic secondary BAs, and diminish DNA damage that fuels TP53, KRAS, and other driver mutations. Neutralizing bacterial genotoxins or deploying phage/CRISPR antimicrobials may selectively eradicate tumor‐promoting taxa. Integrating microbiome emerging GBC therapies may exploit the gut–biliary microbiome to influence mutagenesis and treatment response.

From a therapeutic perspective, decoding the role of individual mutations, and more importantly, the cooperative effects of co‐occurring mutations, is critical for the rational design of targeted therapies in GBC. Our CytoHubba‐based STRING‐derived protein–protein interaction (PPI) network analysis identifies ERBB2 and KRAS as central hub genes, underscoring their functional dominance in GBC signaling architecture and highlighting their potential as dual therapeutic targets (Figure 8). Importantly, the synergistic effect of ERBB2 and KRAS mutations in driving pancreatic ductal adenocarcinoma progression has already been established, where pharmacological co‐targeting with the pan‐mutant ERBB2 inhibitor Neratinib and the KRAS G12C‐specific inhibitor ARS‐1620 yielded profound growth suppression in PDAC xenograft models [135]. By analogy, such strategies could be adapted to GBC, where co‐occurring mutations in ERBB2 and KRAS are increasingly recognized.

**FIGURE 8:**
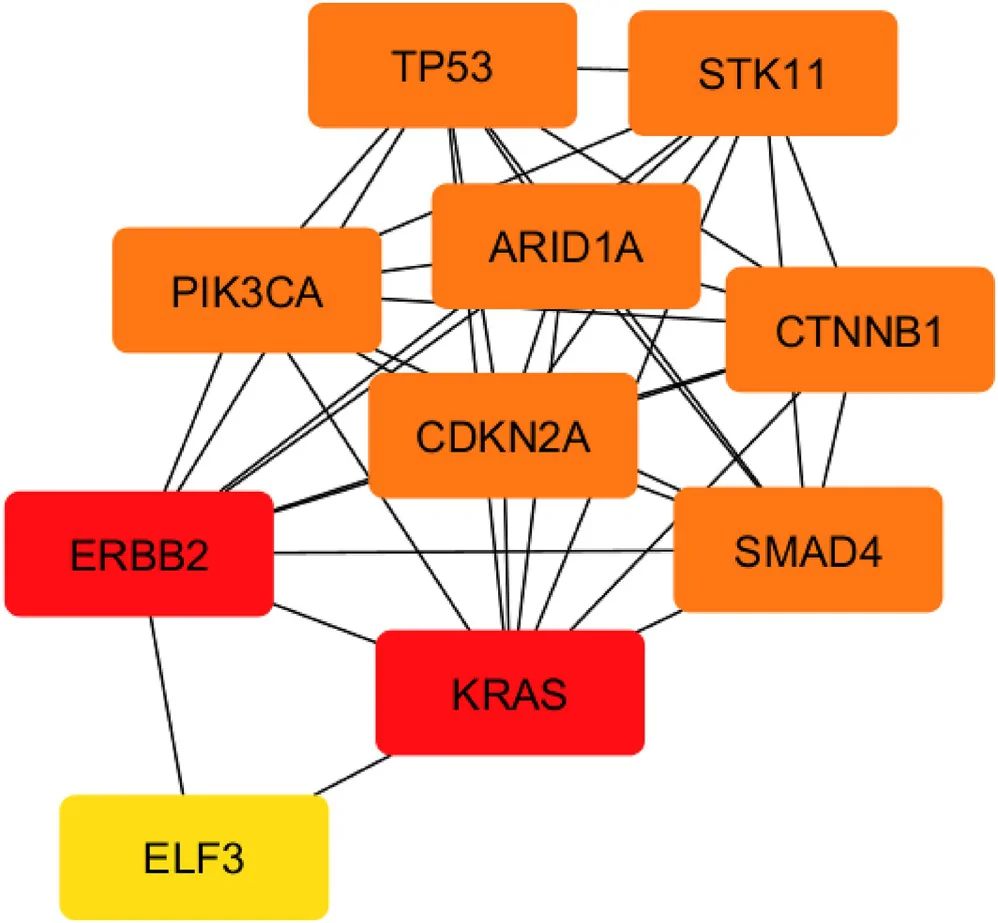
CytoHubba‐based STRING‐derived protein–protein interaction (PPI) network. CytoHubba‐based STRING‐derived protein–protein interaction (PPI) network analysis of 12 studied mutated proteins (TP53, SMAD4, PIK3CA, CDKN2A, ARID1A, ARID2, KRAS, ELF3, ERBB3, ERBB2, STK11, CTNNB1) identified ERBB2 and KRAS as central hub genes, suggesting their functional dominance in GBC.

Advancing our understanding of the functional impact of key mutations and their combinatorial effects on cellular signalling is pivotal for developing effective, personalized therapeutic strategies in GBC. A multi‐omics approach, integrating genomics, transcriptomics, and proteomics, should be utilized to construct comprehensive mutation to phenotype maps. Incorporating mutational profiling into clinical practice promises to transform treatment paradigms from empiric, nonspecific regimens to rationally designed targeted therapies tailored to each patient's unique molecular signature. Exploration of rational drug combinations tailored to specific co‐mutation patterns in GBC should be prioritized, with rigorous preclinical validation using GBC organoids and patient‐derived xenografts. Continued translational research, supported by comprehensive preclinical models and innovative trial designs, will be essential for realizing the full potential of mutation‐specific drug combinations, ultimately improving outcomes for patients with this aggressive malignancy.

## Author Contributions

**Nisha Manav:** conceptualization, data curation, writing – original draft, writing – review and editing, visualization, formal analysis, investigation. **Akanksha Kashyap:** formal analysis, visualization, writing – review and editing. **Lakshay Malhotra:** formal analysis, writing – review and editing, visualization. **Chethan Rajegowda:** formal analysis, writing – review and editing. **Chandra Prakash Prasad:** conceptualization, data curation, formal analysis, visualization, funding acquisition, writing – original draft, writing – review and editing, supervision.

## Funding

This work was supported by the Indian Council of Medical Research (ICMR) [IIRPSG‐2024‐01‐02256 chandraprakashprasad], New Delhi, India.

## Conflicts of Interest

The authors declare that they have no known competing financial interests or personal relationships that could have appeared to influence the work reported in this paper.

## Data Availability

The data that support the findings of this study are available from the corresponding author upon reasonable request.
